# Instrumentation issues in implementation science

**DOI:** 10.1186/s13012-014-0118-8

**Published:** 2014-09-04

**Authors:** Ruben G Martinez, Cara C Lewis, Bryan J Weiner

**Affiliations:** Department of Psychology, Virginia Commonwealth University, 806 West Franklin St., Richmond, VA 23220 USA; Department of Psychological and Brain Sciences, Indiana University, 1101 E. 10th St., Bloomington, IN 47405 USA; Department of Psychiatry & Behavioral Sciences, University of Washington, 1959 NE Pacific Street, Box 356560, Rm BB1644, Seattle, WA 98195-6560 USA; 1102-C McGavran-Greenberg Hall, University of North Carolina at Chapel Hill, 135 Dauer Drive, Campus Box 7411, Chapel Hill, 27599-7411 USA

**Keywords:** Instruments, Implementation, Dissemination, Psychometrics, Mixed methods

## Abstract

**Background:**

Like many new fields, implementation science has become vulnerable to instrumentation issues that potentially threaten the strength of the developing knowledge base. For instance, many implementation studies report findings based on instruments that do not have established psychometric properties. This article aims to review six pressing instrumentation issues, discuss the impact of these issues on the field, and provide practical recommendations.

**Discussion:**

This debate centers on the impact of the following instrumentation issues: use of frameworks, theories, and models; role of psychometric properties; use of ‘home-grown’ and adapted instruments; choosing the most appropriate evaluation method and approach; practicality; and need for decision-making tools. Practical recommendations include: use of consensus definitions for key implementation constructs; reporting standards (*e.g.*, regarding psychometrics, instrument adaptation); when to use multiple forms of observation and mixed methods; and accessing instrument repositories and decision aid tools.

**Summary:**

This debate provides an overview of six key instrumentation issues and offers several courses of action to limit the impact of these issues on the field. With careful attention to these issues, the field of implementation science can potentially move forward at the rapid pace that is respectfully demanded by community stakeholders.

**Electronic supplementary material:**

The online version of this article (doi:10.1186/s13012-014-0118-8) contains supplementary material, which is available to authorized users.

## Background

For centuries it has been said that, ‘science is measurement’ [1], which raises the question: Is measurement necessarily scientific? In the case of new fields such as implementation science, the answer is often ‘no’ [2]. A number of instrumentation issues could threaten the strength of implementation science’s developing knowledge base. A paradox has emerged whereby researchers appear to be investigating implementation initiatives with instruments that may not be psychometrically sound. However, in order to draw conclusions from data and confidently generalize findings, instruments must consistently measure what they are purported to measure—a test only strong psychometrics can affirm [3,4]. It is possible that the demand for the implementation of evidence-based practices (EBPs) may outpace the science if instrumentation issues are not addressed in a principled manner [2,5]. One consequence of these instrumentation issues is that implementation strategy effectiveness cannot yet be easily understood [6]. Without careful attention to these issues, implementation science faces the risk of constructing ‘a magnificent house without bothering to build a solid foundation’ [7,8].

The purpose of this debate is to discuss the following six critical instrumentation issues and to provide recommendations for limiting their impact on implementation science: use of frameworks, theories, and models; role of instrument psychometric properties; use of ‘home-grown’ and adapted instruments; choosing the most appropriate evaluation method and approach; practicality; and need for decision-making tools. Practical and methodological recommendations are provided. Interested readers may refer to Additional file 1 to learn behavioral health-focused implementation researcher perspectives on these issues.

## Discussion

### Instrumentation issue #1: use of frameworks, theories, and models

#### Issue

The growing number of models and diversity of construct definitions may promote similar measurement of disparate constructs or unique measurement of synonymous constructs, making it difficult to report findings in a common language [5,9–11] and/or compare findings across studies [6,12].

### Overview

Implementation research is best conducted when guided by theory [10,12]. Theory and measurement are reciprocally related. Theory defines the content of a construct and describes the relation among constructs. Measurement of constructs can then help to revise and refine theory development. Tabak and colleagues identified over 60 relevant models that characterize the dissemination and implementation process [12]. The panoply of models reflects a growing evidence base [13] and requires careful operationalization of constructs. Each model has a unique structure and varying foci, incorporates variable constructs, and delineates distinct construct definitions [5,14]. Although many implementation science models demonstrate considerable overlap, very few articles aid researchers in demystifying the literature landscape [12]. The Consolidated Framework for Implementation Research (CFIR) is a meta-theoretical framework generated to address the lack of uniformity in the implementation science theory landscape, minimize overlap and redundancies, separates ideas that had been formerly seen as inextricable, and create a uniform language for domains and constructs [15]. However, neither the CFIR nor other existing resources explicitly state how construct definitions diverge between frameworks, models, and theories. This may lead to confusion when determining which model and which instruments to use.

This issue is also highlighted because the use of divergent models can directly impact measurement. Two likely consequences are: models define different constructs the same way (*i.e.*, different terms, same content; synonymy), which yields the same items for measuring ‘different things,’ or models define the same construct in different ways (*i.e.*, same term, different content; homonymy), which gives rise to the use of different items for measuring the ‘same thing.’ These problems reflect linguistic ambiguity, conceptual ambiguity, or both.

Without a consensus language or careful construct operationalization, the instrument’s construct validity and cross-study comparisons of results may be compromised [3,9,16]. For example, the construct of appropriateness is used synonymously with perceived fit, relevance, compatibility, suitability, usefulness, and practicability [17]. These constructs may be conceptualized as the ‘same’ across research teams. However, results from Chaudoir *et al.*’s recent systematic review of implementation instruments at the item level indicate that unique items (*i.e.*, different content) are used to measure these different constructs [18]. Therefore, these constructs may actually represent nuanced, unique factors.

### Recommendations

To build the implementation science knowledge base, identification of key constructs associated with succinct, theoretically informed definitions is critical. Researchers are encouraged to embed investigations in a theoretical framework that will allow a test of predictors, moderators, and mediators of the implementation process and outcomes. Despite the rapid growth of implementation science, it remains unclear which factors are critical for successful implementation, in part because of inadequate and inconsistent use of theory, terminology, and measurement. Tabak *et al*.’s [12] review has importantly positioned researchers to critically engage theory and determine which implementation strategies work when, for whom, and under what conditions.

Consensus terms and definitions may eliminate redundancies in instrument development (issue #6) and build cumulative knowledge [11]. The CFIR wiki (*i.e.*, ‘a site that can be modified or contributed to by users’ [19]) is a coordinated effort that encourages researchers (‘users’) to establish and refine implementation-specific terms and definitions, including specific examples of how constructs are operationalized in the extant literature [20]. The CFIR Wiki presents numerous advantages, as it allows for ongoing communication among researchers, which is critical to the field’s rapid development. Clear definitions, such as those available on the CFIR Wiki, may facilitate researchers’ selection of appropriate instruments for constructs under investigation.

Although the CFIR is relatively comprehensive, the framework does not include implementation outcomes. Moreover, the CFIR is not a theory (*i.e.*, it does not hypothesize interrelations among constructs). For a comprehensive theory of implementation, readers may wish to consider the general theory of implementation proposed by May [21]. Although there may be benefit to endorsing a single conceptual model for use in implementation science, there are also inherent disadvantages to settling on a unifying theory early in a field’s development (*e.g.*, limits discovery, overlooks understudied constructs). At a minimum, researchers are encouraged to include construct definitions to promote transparency of their work and generalizability of their findings.

### Instrumentation issue #2: need to establish instrument psychometric properties

#### Issue

Unless instruments’ psychometric properties are evaluated, confidence cannot be placed in study findings and/or interpretations.

### Overview

Psychometric validation of instruments is arguably among one of the most important aspects of developing a strong empirical foundation for any field [3,22]. Despite this, psychometrics are frequently absent from implementation science articles [3,23]. Chaudoir *et al.*’s review revealed that only 48.4% of the identified instruments reported on the criterion-related validity of the instruments; their review did not assess whether instruments had established reliability or construct validity [18]. Chor *et al.*’s review of measures purported to predict adoption revealed that only 52.5% exhibited *any* established psychometrics [24]. There are several probable reasons for this de-emphasis on psychometrics, including the field’s nascent state and the challenging nature of the ‘real world’ setting placing demands on researchers. Although practicality of instrumentation is inherently important in implementation science where studies are conducted in the field (issue #5), we argue that these factors should not take priority if it leads to compromising psychometrics. Simply put, the quality of the study depends on the quality of the instrumentation.

### Recommendations for reliability reporting

Reliability can be defined broadly as the consistency of scores obtained from an administered instrument [25]. Reliability assessment is most often focused on measures of internal consistency [26], which demonstrates the extent to which items that propose to measure the same general construct produce similar scores in a particular sample. However, internal consistency is not always the most appropriate or important measure of reliability. Test-retest reliability is critical to evaluate when a construct is not expected to change over time, whereas inter-rater reliability is relevant for instruments by which multiple observers rate a target behavior. Researchers should report on the most appropriate assessment of an instrument’s reliability.

### Recommendations for validity reporting

Although there are many kinds of validity (*e.g.*, construct, content, concurrent, divergent, criterion-referenced), validity can loosely be defined as an instrument’s ability to obtain responses representative of the constructs that the developers intended it to measure [3,4,25]. Validity assessment determines how appropriate and useful an instrument is for use in a given setting or interpretation [4]. Validity assessment is touted as ‘the most important consideration in test evaluation’ [4].

The first step to establishing construct validity is carefully defining the construct. Researchers might then engage experts in the initial identification of instrument items, assess face validity with the target population, and pilot the instrument with a sample large enough for assessing validity statistically (*e.g.*, through a factor analysis). Whenever possible, structural validity should be assessed and reported to determine whether the assumption of unidimensionality is met or whether multifactorial latent constructs underlie the data. For additional details on how to maximize validity from the beginning stages of instrument development, readers are referred to published resources [4,27–29].

Finally, criterion-related validity is especially important to report in implementation science given the reciprocal relation between instrument validity and theoretical frameworks. Theoretical frameworks specify hypothesized relations among constructs, and information on concurrent and predictive validity can be used to evaluate and inform theorized relations to refine the theories that guide implementation science [2]. Unfortunately, there remains a dearth of literature delineating the predictive validity of instruments [18]. Building in opportunities to evaluate the impact of factors on the success of an implementation is perhaps one of the most critical understudied areas in implementation science.

### General reporting standards

Reliability and validity are viewed as the most basic and necessary psychometric properties that allow for accurate interpretation of data [3,4,29]. Implementation studies employing instruments without establishing these two forms of psychometrics should alert readers to interpret findings with caution. We are not discouraging the use of instruments that do not have robust psychometrics; indeed, this is a necessary step toward establishing an instrument’s psychometric quality for a given use. A bottom-up process, referred to as epistemic iteration or knowledge acquisition, is important [30]. Through repeated measurement, wherein researchers utilize developing instruments and report psychometric properties obtained from different samples in different settings, the field can discontinue use of unreliable, invalid instruments and confidently administer psychometrically sound instruments. Finally, journals that publish empirical implementation science articles may wish to follow the lead of psychology, which has established reporting standards for instrument psychometric properties [25].

### Instrumentation issue #3: use of ‘home-grown’ and adapted instruments

#### Issue

Use of ‘home-grown’ and/or adapted instruments without carefully attending to appropriate steps of instrument development or assessing and reporting psychometrics may compromise the portability of implementation outcomes to real-world settings [17].

### Overview

The development of new instruments for implementation science is essential, and when done properly allows for reliable and valid interpretations of data [27]. However, in the fast-paced, high-demand field of implementation science there are numerous constraints (*e.g.*, time, lack of expertise) that force investigators to create ‘home-grown’ instruments, defined as instruments created quickly ‘in house’ to assess a construct in a particular study sample, but without engaging proper test development procedures [17]. Home-grown instruments tend to be appropriate only for one-time use, thereby limiting the capacity for cross-study comparisons.

It can be resource-intensive and challenging to conduct a thorough literature review for relevant, accessible, and practical instruments. Given the interdisciplinary nature of implementation science, the literature landscape is broadly dispersed with relevant instruments emerging from disciplines including sociology, engineering, psychology, etc. [13]. This issue is exacerbated by the fact that, until recently, there has been no systematic effort to identify or evaluate instruments to promote ease of access (issue #6). Further still, systematic reviews demonstrate that few instruments are available to assess structural- and patient-level constructs [18]. An additional challenge that researchers face is the lack of sharing of instruments in developmental stages. Moreover, it appears that some of the strongest instruments with demonstrated predictive validity (*e.g.*, the Organizational Social Context; [31]), are proprietary.

Finally, although the dissemination of generic instrumentation would promote ease of use across studies and cross-study comparisons of findings, dissemination of specific instrumentation may be necessary to accurately predict implementation outcomes. Unfortunately, the latter (specific instrumentation) requires researchers working in other areas to adapt instruments by shortening their length or modifying wording. Ultimately, instrument modification may continue to be necessary, but in many instances authors do not report on how instruments are adapted or how adaptations affect the instrument’s psychometric properties [32].

### Recommendations

To decrease resources allocated to the development of redundant instruments and reduce the dissemination of instruments that are not validated for use in a particular setting, we recommend the following. First, researchers may wish to consider relevant models (*e.g.*, [12,21]) to guide the identification of salient constructs. Second, researchers may consider accessing instrument repositories (*e.g.*, SIRC IRP; GEM; issue #6) or published reviews *e.g.*, [18,24] to identify available instruments or to determine whether instrument development is necessary. If a relevant instrument is identified but needs modification, authors should report exactly how the instrument was adapted (to promote replication and transparency), and report the effect of the adaptation on the instrument’s psychometrics properties. Should relevant instruments not be available, the following steps may serve to guide instrument development [27,33,34].

### Step one: defining the construct

The first step of instrument construction should include carefully defining what the construct is and is not, ideally based on existing theory or available definitions.

### Step two: initial item development

After the construct has been defined, relevant items need to be generated. It is important to leverage the expertise of colleagues when identifying the initial pool of items. Until comparisons of generic and specific instruments reveal incremental predictive validity, we argue for researchers to focus on the development of generically worded items that could be used beyond the study for which it is being developed.

### Step three: initial item administration

Items from the initial pool should be administered to a small, representative sample of respondents to assess face validity, identify missing items, and assess whether the language is appropriate, potentially through a think-aloud technique [35].

### Step four: initial item analysis

Once a response set has been obtained, researchers should remove irrelevant or difficult to understand items.

### Step five: administration with a larger sample

A second administration is critical to assess the psychometric properties of the instrument (issue #2). This sample could be the target sample, could occur in the context of the study, and would be ideally powered to assess reliability and validity of the instrument.

### Step six: creating a report

It is essential that instrument developers create a report detailing the methods by which the instrument was constructed, including information on: normative data (*i.e.*, data that characterizes what is usual in a defined population at a specific time point) and evidence of validity (*e.g.*, construct, criterion, etc.; see issue #2) and reliability (*e.g.*, α values for internal consistency, κ values for inter-rater reliability, etc.; see issue #2). This information will encourage appropriate subsequent use of the instrument [27] and will contribute to a cycle of methodological rigor not consistently seen in implementation science.

### Instrumentation issue #4: choosing the most appropriate evaluation method and approach

#### Issue

Use of one method (*e.g.*, self-report) or one approach (*e.g.*, qualitative, quantitative inquiry) may not be appropriate for the study questions, can lead to method bias, and/or limit the strength and contribution of research.

### Overview

There are numerous methods (*e.g.*, self-report, observation, administrative data) by which investigators can assess outcomes and other constructs in an implementation initiative. Self-report allows researchers to learn participant perceptions (*i.e.*, thoughts and feelings). Observation is a means for collecting observable data. Administrative data can provide low-burden accounts of an organization’s functioning. Three main evaluation approaches exist: qualitative, quantitative, and mixed methods. Quantitative approaches are typically used when theory exists and has led to the development of an instrument (self-report) or method (administrative data) suitable for assessing the construct of interest [36]. Qualitative research is often utilized to develop theory, explore themes, and obtain rich information not captured by the constrained response options of self-report [36]. Mixed methods serve multi-faceted functions (see below in recommendations). In sum, each method or approach is used to address different aims and so should be carefully selected.

Self-report is perhaps the most commonly used method for obtaining data in an implementation initiative. Use of self-report makes good sense given that many salient constructs pertain to perceptions of individuals involved (*e.g.*, barriers, facilitators). Moreover, the advantages of self-report are numerous, namely that they appear to be relatively pragmatic in the absence of existing observational infrastructures [37], and self-report instruments have revealed significant predictors of implementation outcomes such as adoption and fidelity [18]. Unfortunately, the disadvantages of self-report methodology are often overlooked. Self-report is prone to biases such as social desirability, leniency bias, and even an individual’s mood [37,38]. For instance, a meta-analysis suggests that while self-report measures and implicit measures of attitudes are related, factors such as social desirability, degree of introspection from the individual, and spontaneity of responses to the instrument affect the degree of the relation [39]. According to Greenwald *et al.* implicit attitude instruments, such as those utilized in social cognition research (*e.g.*, Harvard Implicit Association Test), appear to capture a unique perspective (*i.e.*, different from self-report), and demonstrate strong predictive validity [40]. Thus, even when perceptions are the focus, self-report instruments may not be the optimal method. Finally, studies have shown that for some key implementation outcomes, such as fidelity to the innovation, self-report tends to provide an overestimate of actual use of the EBP when compared with observation [41]. In sum, we argue for the careful consideration of when to use self-report versus independent observation, administrative data, etc.

Similar to the need to carefully select the instrumentation method, implementation science researchers are charged with the difficult task of selecting between quantitative, qualitative, and mixed methods approaches. Because the field of implementation science is still relatively new, the use of mixed-methods approaches (*i.e.*, combination of both qualitative and quantitative) is encouraged [36,42–44]. Utilizing mixed-methods can provide critical, comprehensive insight into barriers and facilitators of the implementation process [36]. Additionally, use of mixed-methods eliminates shared method variance, a problem attributable to the use of a single measurement approach resulting in skewed results [38]. While mixed-methods can be comprehensive, there are inherent weaknesses, particularly that analyzing qualitative data requires significant time and resources.

### Recommendations

When designing an implementation study, investigators should carefully select a method and approach to data collection that is driven by specific aims, extant literature, quality of existing instruments, and the feasibility of employing the ideal methods and approaches. Self-report measures are appropriate when perceptions are the target, but even so (as in the case of attitudes), observation may be optimal. Certain implementation outcomes (*e.g.*, adoption, penetration, fidelity, sustainability; [17]) may require independent observation for accurate assessment. Researchers should consider their options for diversifying assessment methods, including: multi-informant approaches [45], direct observation [46], as well as administrative [47] and existing data such as those captured within the soon to be ubiquitous electronic health records [48]. To aid in the decision of whether and when to use mixed methods, Palinkas *et al.* [36] provide a useful overview of the structure, function, and process of mixed-methods and document five reasons for their use based on a review of the implementation science literature: to understand the implementation process; to engage in both exploratory and confirmatory research; to examine both the content and context of the implementation; to assess consumer perspectives; and, to offset or compensate for one particular method.

In sum, we argue that no evaluation method or approach is inherently better or worse; rather, researchers should be intentional when deciding how to proceed based on their research questions and the extant literature. For instance, if researchers wish to report on the effectiveness of an intervention they may choose quantitative evaluation strategies that allow for sophisticated statistical analyses. Researchers that intend to perform exploratory research on the barriers to implementing an EBP in a novel setting may utilize qualitative inquiry to gather detail-rich data. Researchers that plan to investigate observable outcomes as well as understand a nuanced aspect of their implementation process may choose to utilize mixed-methods. Although multiple (self-report and observation) and mixed-methods (quantitative and qualitative) may present additional challenges to the evaluation process (*e.g.*, cost, personnel resources, time), careful design may ultimately provide critical insights into the implementation process and remove the disadvantages presented by a single evaluation approach.

### Instrumentation issue #5: practicality

#### Issue

Given that implementation science takes place in real world settings, identifying practical or pragmatic [49] instruments is critical.

### Overview

Both researchers and stakeholders require practical (*e.g.*, accessible) or pragmatic (*e.g.*, actionable) instruments [49]. Unfortunately, practical or pragmatic qualities may not be a top priority in the initial stages of ‘proper’ instrument development [27]. This means that implementation researchers have to carefully construct the instrument battery prioritizing only those constructs and items considered to be critical to evaluate the impact of the implementation. This process often results in a dilemma wherein researchers must choose between instruments that are practical versus those with validated psychometrics.

### Recommendations

Given the need for ongoing instrument development in implementation science, instrument developers might wish to consider the following four categories of practicality.

### Cost

It is sometimes the case that developers create proprietary instruments. While it is understood and appreciated that a great deal of work goes into the creation and psychometric validation of these instruments, it may be important for instrument developers to avoid commercialization to move implementation science forward.

### Accessibility

Although researchers creating ‘home-grown’ instruments (issue #4) might not have had an adequate sample size to establish the instrument’s psychometric properties (issue #2), researchers might still share their instrument in an existing repository (issue #6) or in the publication summarizing their work to enable others to contribute to the evidence base.

### Length

Developers should be conscious of the instrument length to promote use in resource-demanding settings. Several validated instruments tapping pertinent implementation science constructs include hundreds of items (per construct). Thus, even though it is desirable to assess more than one construct in an implementation evaluation, it is typically impractical for researchers or stakeholders to administer such time-consuming instruments. An additional advantage to creating shorter instruments is that of minimizing respondent errors due to ‘fatigue and carelessness’ [38].

### Language

The use of common or easy-to-understand language is key to instrument practicality. Complex language or ambiguity of items can cause respondent error, potentially leading to skewed results [38]. Developers should follow guidelines set forth by Walsh and Betz [27], including piloting instruments with a representative group.

Finally, Glasgow and Riley recently put forth criteria for ‘pragmatic’ behavioral health measures [49]. Specifically, Glasgow and Riley [49] state that instruments (measures) should be important to stakeholders, low burden, actionable, and sensitive to change. We argue that these pragmatic qualities may also be important for implementation-specific instruments. Stakeholders may wish to use implementation instruments to prospectively assess organizational needs and contexts (to select implementation strategies), monitor implementation strategy impacts, and refine implementation processes to optimize outcomes. Attending to these qualities throughout the development and testing process could increase the utility of instruments to advance the science and practice of implementation.

### Instrumentation issue #6: need for decision-making tools

#### Issue

Despite the relatively young state of implementation science, there are many instruments available, making the need for decision tools and repositories a priority.

### Overview

As a result of the issues discussed above, decision-making tools are needed to elucidate the quality and array of implementation science instruments available. It is clear that the expansive interdisciplinary literature landscape, though more easily navigable given recent systematic reviews [18,24], remains somewhat elusive and overwhelming for researchers. To aid researchers in building the foundation of implementation science based on robust instrumentation, repositories equipped with decision tools are critical.

### Recommendations

Largely in response to the issues raised throughout this debate, teams from the NIMH-funded SIRC IRP [50] and the National Cancer Institute (NCI)-funded Grid-Enabled Measures project (GEM) [51] have attempted to identify and categorize implementation science instruments. These teams endeavor to disseminate valid, reliable, and pragmatic instruments.

The SIRC IRP, supported in kind by National Institutes of Mental Health (NIMH), employs a multi-faceted, collaborative rigorous methodology that attempts to compile, organize, and empirically rate instruments tapping the CFIR [15] and implementation outcomes constructs [17]. The SIRC IRP will be available to SIRC members^a^ and aims to assist researchers in identifying relevant, psychometrically validated, and practical instruments. The SIRC IRP methodology produces head-to-head graphical comparisons of psychometric properties for all available instruments to serve as a decision-aid for researchers.

The NCI GEM Project is a collaborative web-based tool with the goal of ‘supporting and encouraging a community of users to drive consensus on best measures and share the resulting data from use of those measures’ [51]. The GEM project allows users to add their own constructs and definitions, upload their instruments, and give instruments a rating from one to five stars to promote comparison and selection based on validity, reliability, and pragmatic properties. Ultimately, each team seeks to create decision-making tools for optimal instrument selection to promote the ease with which researchers can engage methodologically rigorous evaluation.

### Summary

A number of instrumentation issues have been raised that potentially threaten the methodological rigor of a promising field. This debate presented specific issues in hopes of promoting careful consideration of how to limit the effect of these issues on the field. Recommendations included reporting standards, a succinct guide to instrument development, and decision aids for researchers to engage. Table 1 depicts a concise summary of the identified issues and recommendations. Ultimately, through this article, implementation researchers might be more equipped to think critically about instrument development and administration, the factors influencing the quality of instrumentation, the limitations and strengths of different instrumentation methods and evaluation approaches, and which instruments possess adequate psychometric properties. The fact remains that without psychometrically validated instruments, investigators cannot be confident that instruments measure the purported constructs consistently. It is hoped that the recommendations provided will lead to improvements in implementation science evaluation.

**Table 1:** **Overview of instrumentation issues and recommendations**

**Issue**	**Recommendation**
1. Use of frameworks, theories, and models	• Use theoretical model and include those construct definitions in manuscripts
	• Work towards consensus language as a field
	• Consider use of the CFIR Wiki for construct definitions
2. Need to establish instrument psychometric properties	• Identify, perform, and report results of most appropriate reliability assessments when possible
	• Identify, perform, and report results of most appropriate validity assessments when possible
	• Attempt to establish psychometric properties while simultaneously investigating factors involved in the implementation process *e.g.*, [52]
3. The use of ‘home-grown’ and adapted instruments	• Utilize SIRC IRP and NCI GEM projects to identify existing instruments for constructs under evaluation
	• Utilize proper test development procedures [27,33,34]
	• Report adaptations (changes in length, language, structure) and updated psychometrics to assess effect of adaptations
4. Choosing the most appropriate evaluation method and approach	• Consider utilizing mixed-methods approaches
	• When appropriate, consider utilizing multi-informant, direct observation, and administrative data in addition to or instead of self-report
5. Practicality	• Keep instrument costs as low as possible
	• Keep item numbers low (perhaps 10 or fewer)
	• Provide the instrument items in the published manuscript
	• Provide the instrument to the NCI GEM project or SIRC IRP
	• Make the language accessible
6. Need for decision-making tools	• Utilize the SIRC IRP and NCI GEM projects to identify instruments
	• Report on any and all results of psychometric analyses

*Note.* CFIR = Consolidated Framework for Implementation Research [15]; SIRC IRP = Seattle Implementation Research Collaborative Instrument Review Project; NCI GEM = National Cancer Institute Grid-Enabled Measures Project.

### Endnote

^a^Interested readers can register for SIRC membership at the following webpage: http://www.societyforimplementationresearchcollaboration.org/.

## Additional file

Additional file 1:
**This file contains a survey regarding instrumentation issues that was distributed to the Society for Implementation Research Collaboration (SIRC) Network of Expertise and the Association of Behavioral and Cognitive Therapies Dissemination and Implementation Science Special Interest Group (ABCT DISSIG) listservs.** Additionally, information on the creation of the instrument, demographic information of the respondents, and a summary of the results are provided [2,12,15,53–68].

## Abbreviations

CFIR: Consolidated framework for implementation research
EBP: Evidence-based practice
GEM: Grid-enabled measures project
NCI GEM: National cancer institute grid-enabled measures project
NIMH: National institutes of mental health
SIRC: Society for implementation research and collaboration
SIRC IRP: Society for implementation research and collaboration instrument review project
