# Supplementary material for: Instrumentation issues in implementation science
Source: Implement Sci. 2014 Sep 4;9:118. doi: 10.1186/s13012-014-0118-8 (PMC4164742; doi:10.1186/s13012-014-0118-8)
Supplement: Additional file 1: — This file contains a survey regarding instrumentation issues that was distributed to the Society for Implementation Research Collaboration (SIRC) Network of Expertise and the Association of Behavioral and Cognitive Therapies Dissemination and Implementation Science Special Interest Group (ABCT DISSIG) listservs. Additionally, information on the creation of the instrument, demographic information of the respondents, and a summary of the results are provided [2,12,15,53–68]. [file 13012_2014_118_MOESM1_ESM.docx]

**Gathering Perspectives**

To expand the perspectives reflected in this debate, a survey (survey and descriptive statistics found below) was designed to engage researchers in determining the relative importance and priority of instrumentation issues in implementation science. Initial issues were generated by the authors based upon literature review as well as lessons learned through the Society for Implementation Research Collaboration (SIRC) Instrument Review Project (IRP; see http://www.seattleimplementation.org/sirc-projects/sirc-instrument-project/). Issues generated by the authors were refined by SIRC core research team members. The survey contained 14 questions, three of which pertained to respondents’ experience with implementation science (e.g., years in the field) and 11 pertained to respondents’ experience with each of the issues. Questions related to the latter focused on use of mixed methods and theoretical models, methods of instrument development, and perceptions of issue importance. Respondents were given the option to provide unique responses. The survey can be found below.

Participants were identified via two implementation science-related listservs: the SIRC Network of Expertise (N=117) and the Association of Behavioral and Cognitive Therapies Dissemination and Implementation Science Special Interest Group (N=212). A total of 329 people received the request to complete the online survey and 81 responses were received. On average, participants reported 10 years (SD=8.6) of implementation experience and 8.7 (SD=13) implementation projects completed. Ninety percent (N=73) of respondents self-identified as researchers, 27% as practitioners, 7% as administrators, and 4% as purveyors. The majority of respondents reportedly conducted implementation projects in community mental health (47%), specialty mental health (27%), and school (26%) settings.

Several limitations regarding the data and perspectives reported herein are important to note. First, the survey garnered only a modest response rate of 24.6%. Second, the survey data reflects only those perspectives of self-selected members from two associations that primarily include behavioral health implementation researchers. Third, the survey was overly simplified to reduce burden and to confirm/disconfirm the authors’ perceptions of instrumentation issue priorities. These limiting factors suggest the results may represent a point of view specific to a subset of researchers and the recommendations should be interpreted with this in mind.

.

**Instrumentation Issues survey/request for completion distributed to listservs**

We are writing to invite you to complete a survey on Measurement Issues in Implementation Science. We are looking to identify what implementation scientists believe are the key measurement issues implicated in our field. Data from this survey will inform a paper the Seattle Implementation Research Conference Instrument Review Task Force is writing. There is no obligation to participate and you may choose to discontinue your participation at any time without penalty. There is no compensation for your participation, though we hope the field of implementation science will benefit from raising awareness on some of these issues. No identifying information will be collected through this survey. The survey takes roughly 20 minutes to complete. Thank you very much for your time and participation, and please let contact us if you have any questions! Dr. Cara C. Lewis (lewiscc@indiana.edu) & Ruben Martinez (rgm@indiana.edu)

IRB Study # 1212010257 - Organizations that may inspect and/or copy your research records for quality assurance and data analysis include groups such as the study investigator and his/her research associates, the Indiana University Institutional Review Board or its designees, and (as allowed by law) state or federal agencies, specifically the Office for Human Research Protections (OHRP) who may need to access your research records. For questions about your rights as a research participant or to discuss problems, complaints or concerns about a research study, or to obtain information, or offer input, contact the IU Human Subjects Office at (812) 856-4242 or by email at irb@iu.edu Taking part in this study is voluntary.  You may choose not to take part or may leave the study at any time.  Leaving the study will not result in any penalty or loss of benefits to which you are entitled.  Your decision whether or not to participate in this study will not affect your current or future relations with

**Survey & Descriptive Statistics**

**Q1** How would you characterize your stakeholder involvement with dissemination and implementation (D&I) science?  D&I can be defined as research that intends to bridge the gap between clinical research and everyday practice by building a knowledge base about how mental health care information and new practices are transmitted and translated for health care service use in specific settings. Source: <http://grants.nih.gov/grants/guide/pa-files/pa-02-131.html>. Please check all that apply.

| **Stakeholder Involvement** | ***f* (%)** |
| --- | --- |
| Researcher (University) | 50 (61.7%) |
| Practitioner | 22 (27.2%) |
| Researcher (Non-profit) | 18 (22.2%) |
| Researcher ( For-profit) | 5 (6.2%) |
| Administrator (management) | 7 (8.6%) |
| Student | 3 (3.7%) |
| Purveyor | 3 (3.7%) |
| Policy Maker | 0 (0.0%) |
| Other (18): Student, Implementation Capacity Development | |

**Q2** How many years have you been a stakeholder in the field of dissemination & implementation? Please enter a number in the box below.

| **M (SD)** | **Range** |
| --- | --- |
| 10.2 (8.6) | 42 (0-42) |

**Q3** In what setting do you conduct the majority of your implementation efforts? Please check all that apply.

| **Setting** | ***f* (%)** |
| --- | --- |
| Community mental health | 38 (46.9%) |
| Specialty mental health clinic | 23 (28.4%) |
| School | 21 (25.9%) |
| Primary care | 19 (23.5%) |
| Children’s services (including foster care and CPS) | 16 (19.8%) |
| Veteran’s Affairs | 15 (18.5%) |
| Community organization/non-profit/NGO (Not behavioral health treatment) | 14 (17.3%) |
| Public health | 9 (11.1%) |
| Small for-profit business | 6 (7.4%) |
| Substance abuse treatment | 6 (7.4%) |
| Department of Corrections | 4 (4.9%) |
| Inpatient psychiatry | 3 (3.7%) |
| Inpatient medical | 3 (3.7%) |
| Specialty medical-surgical | 3 (3.7%) |
| Large for-profit business | 2 (2.5%) |
| Department of Defense | 0 (0.0%) |
| Other (3): No responses provided | |

**Q4** The following are the most important measurement issues facing implementation science: If no response for "Other" please leave blank.

**Q5** Please rank the order of the importance of these issues with regard to measurement in implementation science.  If you do not have any additional issues to add in "other", please leave "other" options blank at 10-13.  Drag & Drop to change an issue's rank.  If no responses for "other", please leave them in slots 14-16.

| **Instrumentation Issues** | | |
| --- | --- | --- |
| **Identified Issue** | **Rank**  ***M*  (*SD*)** | **Likert**  ***M* (*SD)*** |
| 1. Psychometric Issues ’(i.e. use of "proper" test development) | 4.45 (2.4) | 3.82 (.97) |
| 2. Theoretical frameworks to guide the implementation process and operationalize constructs | 4.55 (3.59) | 4.01 (.91) |
| 3. Need for communication within the field with respect to instrumentation | 5.36 (2.78) | 4.12 (.74) |
| 4. Lack of short and user-friendly instrumentation | 5.46 (3.69) | 4.30 (.92) |
| 5. Lack of instruments for important constructs relevant to D&I | 5.58 (4.15) | 4.26 (.87) |
| 6. Lack of decision-making tools for choosing appropriate and psychometrically sound instruments | 6.78 (3.7) | 4.03 (.90) |
| 7. Necessary adaptation of existing instruments | 7.69 (2.54) | 3.65 (.85) |
| 8. ‘’Homegrown’’ instruments | 7.70 (3.14) | 3.64 (1.05) |
| 9. Over-reliance on self-reports | 7.87 (3.63) | 3.74 (1.05) |
| 10. Lack of consensus battery | 8.09 (4.27) | 3.74 (1.11) |
| 11. Lack of or inappropriate use of mixed-method instrumentation | 8.52 (3.11) | 3.57 (1.02) |
| 12. Limited use of archival and objective data | 10.01 (3.22) | 3.5 (.94) |
| 13. Unnecessary adaptation of existing instruments | 10.12 (2.38) | 3.01 (.87) |

*Note.* Table corresponds to Q4 and Q5. Respondents ranked issues from 1=most important to 10=not important at all. Respondents also endorsed whether they “Strongly disagree that this is an issue” (score of 1) to “Strongly agree that this is an issue” (score of 5). The two columns suggest relative and degree of importance of each issue, respectively.

**Q6** How many distinct D&I projects have you been involved in, at any stakeholder level? Please enter a number in the box below.

| **M (SD)** | **Range** |
| --- | --- |
| 8.65 (13.17) | 100 (0-100) |

**Q7** Of the D&I projects that you have been involved in, how many times did you use an established theory or framework to guide the implementation process & operationalize constructs? Please enter a number in the box below.

| **M (SD)** | **Range** |
| --- | --- |
| 5.29 (10.78) | 80 (0-80) |

**Q8** If so, which of the following have you utilized?  Select all choices that apply.  If none of the choices below are accurate, please add in "Other".

| **Models** | | |
| --- | --- | --- |
| **Model Name** | **D vs. I focus [**[**12**](#_ENREF_12)**]** | **% sample used** |
| Diffusion of Innovation Theory [[52](#_ENREF_52)] | D-only | 32% |
| Conceptual Framework for Research, Knowledge Transfer, & Utilization [[53](#_ENREF_53)] | D-only | 2% |
| Framework for Knowledge Translation [[54](#_ENREF_54)] | D-only | 6% |
| A Conceptual Model for the Diffusion of Innovations in Service Organizations [55] | D>I | 15% |
| Framework for Dissemination of Evidence-Based Policy [56] | D>I | 5% |
| A Framework for Analyzing Adoption of Complex Health Innovations [[5](#_ENREF_55)7] | D>I | 1% |
| RE-AIM Framework [58] | D=I | 17% |
| Interactive Systems Framework [59] | D=I | 11% |
| “4E" Framework for Knowledge Dissemination and Utilization [60] | D=I | 3% |
| Facilitating Adoption of Best Practices (FAB) Model [[61](#_ENREF_61)] | I>D | 3% |
| The Practical, Robust Implementation and Sustainability Model [62] | I>D | 3% |
| Pathways to Evidence Informed Policy [63] | I>D | 0% |
| Consolidated Framework for Implementation Research [[15](#_ENREF_15)] | I-only | 28% |
| The Active Implementation Framework [64] | I-only | 26% |
| Implementation Outcomes [[2](#_ENREF_2)] | I-only | 19% |
| Availability, Responsiveness, & Continuity: An Organizational and Community Intervention Model [[65](#_ENREF_65)] | I-only | 12% |
| Promoting Action on Research Implementation in Health Services [66] | I-only | 7% |
| Other (Most referenced: National Implementing EBP Project Framework; Replicating Effective Programs model) [[67](#_ENREF_67)] | N/A | 15% (3% most referenced) |

*Note.* D = Dissemination. I = Implementation. The survey was populated with models from Tabak et al.’s review [12] taking an equal sampling of D versus I and D = I focused models. An “other” option was provided for respondents to indicate use of models not provided in the standard response options.

**Q9** How many times have you been forced to generate instruments to assess D&I constructs central to your project due to the fact that no established instruments existed? Please enter a number in the box below.

| **M (SD)** | **Range** |
| --- | --- |
| 4 (3.97) | 20 (0-20) |

**Q10** Which constructs have you been forced to generate instruments for? Please enter constructs below, separated by semicolons.

| **#** | **Construct** |
| --- | --- |
| **1** | Implementation process (pre-implementation, implementation, sustainability) |
| **2** | Implementation milestone achievement |
| **3** | Cost of implementation |
| **4** | Community partnerships |
| **5** | Coalition building |
| **6** | Stakeholder engagement |
| **7** | Community organizing |
| **8** | Sustainability |
| **9** | Family activation/engagement |
| **10** | Financial viability |
| **11** | Organizational readiness to adopt |
| **12** | Exploration stage assessment (problems, solutions, resources, fit, current implementation strength/gaps) |
| **13** | Installation stage assessment (people, skills, organization preparation to do the work of implementation) |
| **14** | Implementation drivers (extent to which best practices are in place for developing practitioner competencies, supporting implementation and intervention capacity, and providing leadership for change and sustainability |
| **15** | Implementation outcomes (fidelity/performance assessment for practitioners and implementation team members |
| **16** | Self-determination in relationship to developmental disabilities |
| **17** | Friendship development in relationship to developmental disabilities |

**Q11** Do you employ mixed-methods instrumentation as part of your implementation process?

| **Yes (%)** | **No (%)** | **Did not answer (%)** |
| --- | --- | --- |
| 14 (17.3%) | 59 (72.8%) | 8 (9.9%) |

**Q12** If yes, for what purpose? The following five functions have been pulled from Palinkas and colleagues' recent paper.  All response options cited from: Palinkas, Lawrence A., Aarons, Gregory A., Horwitz, Sarah, Chamberlain, Patricia, Hurlburt, Michael, & Landsverk, John. (2011). Mixed method designs in implementation research. Administration and Policy in Mental Health and Mental Health Services Research, 38(1), 44-53.Please select all that apply.

| **Purpose (via Palinkas)** | ***f (%)*** |
| --- | --- |
| "Convergence - Using both types of methods to answer the same question, either through comparions of results to see if they reach the same conclusion (triangulation) or by converting a data set from one type into another (e.g. quantifying qualitative data or qualifying quantitative data)" | 30 (37.0%) |
| "Complementarity - Using each set of methods to answer a related question or series of questions for purposes of evaluation (e.g., using quantitative data to evaluate outcomes and qualitative data to evaluate process) or elaboration (e.g. using qualitative data to provide depth of understanding and quantitative data to provide breadth of understanding)" | 44 (54.3%) |
| "Expansion - Using one type of method to answer questions raised by the other type of method (e.g., using qualitative data set to explain results of quantitative data set)" | 26 (32.1%) |
| "Development - Using one type of method to answer questions that will enable use of the other method to answer other questions (e.g., develop data collection measures, conceptual models, or interventions)" | 23 (28.4%) |
| "Sampling - Using one type of method to define or identify the participant sample for collection and analysis of data representing the other type of method (e.g., selecting interview informants based on responses to survey questionnaire)" | 13 (16.0%) |

**Q13** In the case of utilizing instruments to tap a central Dissemination & Implementation construct for your study, do you prioritize psychometric validation or practicality? Please drag line to the appropriate percentage for each.  The total percentage of BOTH lines must equal 100%.

| **Answer** | **Min Value** | **Max Value** | **Mean value (SD)** |
| --- | --- | --- | --- |
| Psychometric Validation | 0 | 90 | 48.17 (16.95) |
| Practicality | 10 | 100 | 51.83 (16.95) |

**Q14** What do you believe renders an instrument optimally practical to use? Please check all that apply.

| **Answer** | ***f* (%)** |
| --- | --- |
| Length | 59 (72.8%) |
| Usability | 56 (69.1%) |
| Practical Language | 55 (67.9%) |
| Psychometric evidence | 54 (66.7%) |
